# Preoperative evaluation to determine the difficulty of No. 6 lymphadenectomy in laparoscopic gastrectomy

**DOI:** 10.1186/s12893-024-02349-8

**Published:** 2024-02-22

**Authors:** Chie Takasu, Masaaki Nishi, Kozo Yoshikawa, Takuya Tokunaga, Hideya Kashihara, Yuma Wada, Toshiaki Yoshimoto, Mitsuo Shimada

**Affiliations:** Department of Surgery, University of Tokushima, 3-18-15 Kuramoto-cho, Tokushima, 770-8503 Japan

**Keywords:** Gastric cancer, Prediction, Pancreatic fistula, Trainee, Laparoscopic gastrectomy, No. 6 LND

## Abstract

**Background:**

Laparoscopic gastrectomy (LG) requires a long learning curve because of the complicated surgical procedures. Infrapyloric (No. 6) lymph node dissection (LND) is one of the difficult procedures in LG, especially for trainees. This study investigated the impact of the prediction of the difficulty of No. 6 LND.

**Methods:**

We retrospectively reviewed the preoperative computed tomography (CT) images and individual operative video records of 57 patients who underwent LG with No. 6 LND to define and predict the No. 6 LND difficulty. To evaluate whether prediction of the difficulty of No. 6 LND could improve surgical outcomes, 48 patients who underwent laparoscopic distal gastrectomy were assessed (30 patients without prediction by a qualified surgeon and 18 patients with prediction by a trainee).

**Results:**

The anatomical characteristic that LND required > 2 cm of dissection along the right gastroepiploic vein was defined as difficulty of No. 6 LND. Of the 57 LG patients, difficulty was identified intraoperatively in 21 patients (36.8%). Among the several evaluated anatomical parameters, the length between the right gastroepiploic vein and the right gastroepiploic artery in the maximum intensity projection in contrast-enhanced CT images was significantly correlated with the intraoperative difficulty of No. 6 LND (*p* < 0.0001). Surgical outcomes, namely intraoperative minor bleeding, postoperative pancreatic fistula, and drain amylase concentration were not significantly different between LG performed by a trainee with prediction compared with that by a specialist without prediction.

**Conclusions:**

Preoperative evaluation of the difficulty of No. 6 LND is useful for trainees, to improve surgical outcomes.

## Introduction

Laparoscopic gastrectomy (LG) is becoming a standard procedure for gastric cancer, and the indications for LG have been extended to advanced gastric cancer [1, 2]. However, LG has several difficult components, especially for trainees, namely complicated surgical procedures, lymph node dissection (LND), and resection of several blood vessels. Infrapyloric (No. 6) lymph node dissection (LND) is one of the difficult procedures in LG because this lymph node directly faces the pancreas and dissection may lead to postoperative pancreatic fistula (POPF). Because the intraoperative procedures during LG may result in pancreatic injury, operator skill [3] and understanding of the local anatomy [4] are necessary for surgical safety. The surgeon is required to identify the pancreatic border without any tactile sensation during LG. Sometimes it is difficult to distinguish the pancreas from fat tissue owing to the rough and irregular surface of the pancreas. Regarding the anatomical characteristics in No. 6 LND, Kobayashi et al. reported that the “process of the pancreas head (PPH),” which defined protruding pancreatic tissue on the anterior side of the pancreas head, is associated with the risk of POPF. The prediction of a PPH using preoperative computed tomography (CT) images was useful to prevent POPF [4]. However, we have often encountered difficulty in No. 6 LND, in addition to that related to the presence of a PPH. Individuals in whom the right gastroepiploic vein (RGEV) lies a long distance from the root to the clipping point in front of the pancreas require prolonged LND along the RGEV. We defined this characteristic as difficulty of No. 6 LND. Previously, we inadvertently injured the pancreas during No. 6 LND perioperatively in cases with such difficulty, resulting in irreversible damage to the pancreas.

In this study, we aimed to investigate the impact of prediction of the difficulty of No. 6 LND, to improve surgical outcomes in LG, especially for trainees.

## Materials and methods

### Patients

Patients preoperatively diagnosed with gastric cancer who underwent LG with No. 6 LND at Tokushima University Hospital from January 2016 to December 2021 were enrolled in this study. We retrospectively reviewed the preoperative computed tomography (CT) images and individual operative video records of 57 patients who underwent LG with No. 6 LND to define and predict the difficulty of No. 6 LND. To evaluate whether prediction of the difficulty of No. 6 LND could improve surgical outcomes, 48 patients (30 patients without prediction by a qualified surgeon and 18 patients with prediction by a trainee) who underwent laparoscopic distal gastrectomy (LDG) were assessed using clinicopathological and surgical factors. Each participant provided written informed consent for inclusion in the study, which was authorized in advance by the Institutional Review Board of the University of Tokushima Graduate School (No. 3215-2).

### Definition of intraoperative difficulty of No. 6 LND

**Fig. 1:**
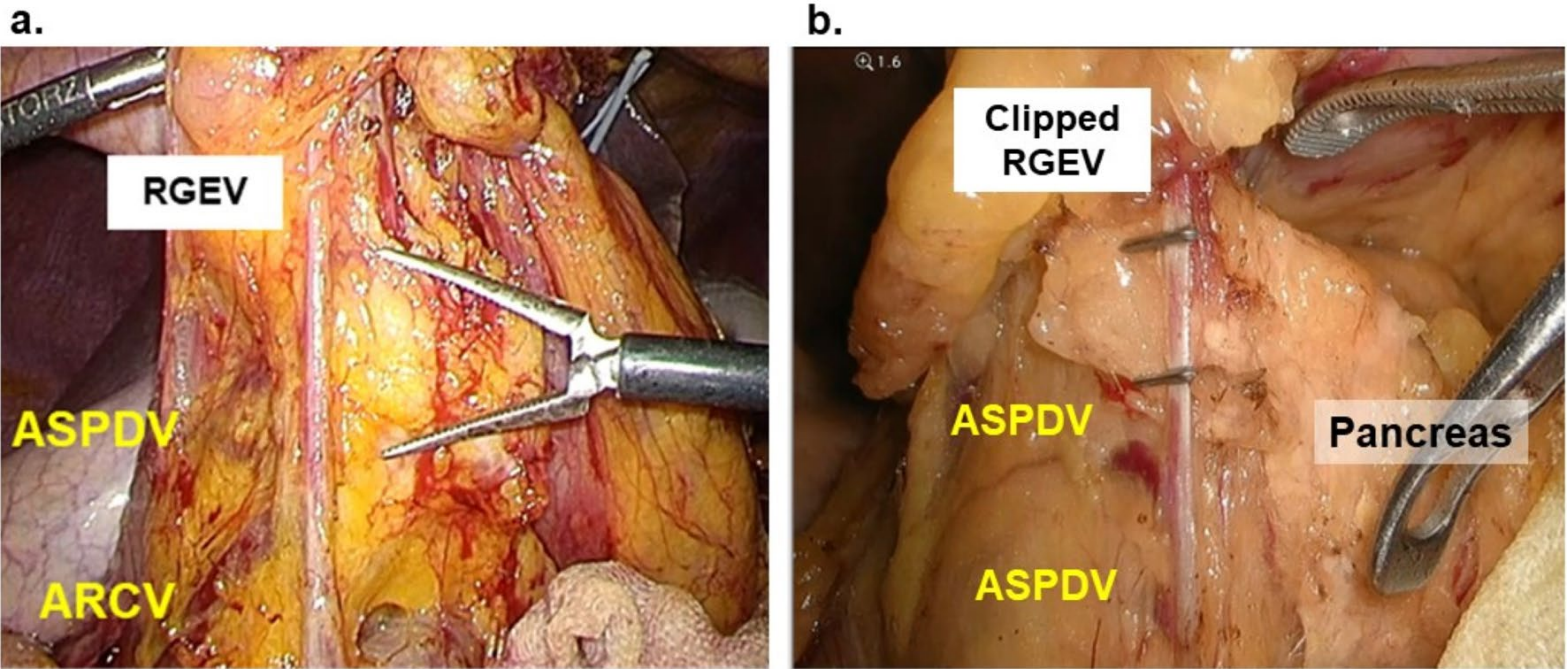
Intraoperative images of a difficult case of No. 6 lymph node dissection. Two representative images of a difficult case are shown (**a**, **b**). The top of the pancreas was far from the root of the RGEV, and longer LND was required than in an average case. We defined this characteristic, i.e., dissection along the RGEV required a distance of > 2 cm, as difficulty of No. 6 LND. *RGEV* right gastroepiploic vein, *ASPDV* anterior superior pancreaticoduodenal vein, *ARCV* accessory right colic vein, *LND* lymph node dissection

The presence of intraoperative difficulty of No. 6 LND was evaluated by viewing individual operative video records. In accordance with a previous report of the procedure for LG, No. 6 LND began with opening the omental bursa [5]. The division proceeded rightward beyond the right border of the omental bursa to the lower edge of the descending part of the duodenum. Generally, the roots of the RGEV and the anterior superior pancreatic duodenal vein were exposed, and the origin of the RGEV was divided by clipping. No. 6 LND proceeded from this point upward. The raised outline along the right side of the RGEV was carefully exposed to dissect the No. 6 LND from the anterior surface of the pancreas. Maintaining the dissection along the top of the pancreas, the right gastroepiploic artery (RGEA) was identified and divided by clipping. However, in certain cases, the top of the pancreas was far from the root of the RGEV, and prolonged LND was required. We defined this characteristic of the dissection along the RGEV requiring > 2 cm from the root of the RGEV, as difficulty of No. 6 LND. Two representative images of a difficult case are shown (Fig. 1a, and b). Because identifying the layer between the fat and the pancreas is important to avoid pancreatic injury, the risk of pancreatic injury is increased in difficult LND cases.

### Evaluation of the anatomical characteristics using CT images

**Fig. 2:**
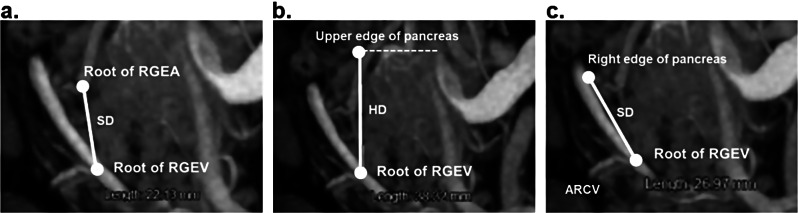
Representative MIP images of the anatomical parameters. (**a**) Measurement of the slope distance between the root of the RGEV and the RGEA (**b**) Measurement of the horizontal distance between the points of the upper edge of the pancreas and the root of the RGEV (**c**) Measurement of the slope distance between the right edge of the pancreas where the RGEV passes through and the root of the RGEV. *MIP* maximum intensity projection, *RGEV* right gastroepiploic vein, *RGEA* right gastroepiploic artery

We retrospectively reviewed the patients’ preoperative contrast-enhanced abdominal CT images to investigate the parameters that predict the difficulty of No. 6 LND. We preliminary measured 13 parameters (Table 1) with 10 patients characterized as difficult No. 6 LND using the anatomical features around the No. 6 lymph node. Representative images of these parameters using the maximum intensity projection (MIP) are shown in Fig. 2. Validation was subsequently performed using all patients’ data.

**Table 1:** Anatomical parameters using preoperative CT images

Variables	Difficulty (-) (*n* = 10)	Difficulty (+) (*n* = 10)	p-value
**Axial plane**			
Ventral edge of SMV– ventral edge of pancreas: HD	28.6 ± 8.6	30.0 ± 7.6	0.79
Right edge of SMV– right edge of pancreas: HD	4.6 ± 4.2	9.1 ± 4.6	0.15
Root of GCT - edge of pancreas where RGEV pass through: SD	17.7 ± 16.0	18.4 ± 13.5	0.95
Root of RGEV – root of RGEA: HD	8.1 ± 5.3	13.3 ± 11.6	0.43
Root of RGEV – root of RGEA: SD	8.8 ± 5.6	9.4 ± 3.6	0.83
Root of RGEV - edge of pancreas where RGEV pass through: SD	9.4 ± 11.4	17.4 ± 7.0	0.20
**Coronal plane**			
Right edge of SMV– right edge of pancreas: HD	17.3 ± 5.5	18.8 ± 14.5	0.85
Root of GCT – right edge of pancreas: HD	29.8 ± 10.9	30.1 ± 5.8	0.96
Root of RGEV – root of RGEA: SD	16.6 ± 4.8	26.6 ± 7.7	0.06
Root of RGEV – root of RGEA: HD	14.1 ± 4.9	25.4 ± 7.8	**0.04**
**MIP**			
Root of RGEV – root of RGEA: SD	15.3 ± 3.0	26.0 ± 7.2	**0.02**
Root of RGEV – Upper edge of pancreas: HD	15.0 ± 3.4	32.6 ± 8.4	**0.005**
Root of RGEV – edge of pancreas where RGEV pass through: SD	13.9 ± 10.1	24.1 ± 16.3	0.30

*CT* computed tomography, *HD* horizontal distance, *SD* slope distance, *SMV* superior mesenteric vein, *GCT* gastrocolic trunk, *RGEV* right gastroepiploic vein, *RGEA* right gastroepiploic artery, *MIP* maximum intensity projection

The bold values means significant difference (p<0.05)

### Surgical and postoperative outcomes

**Fig. 3:**
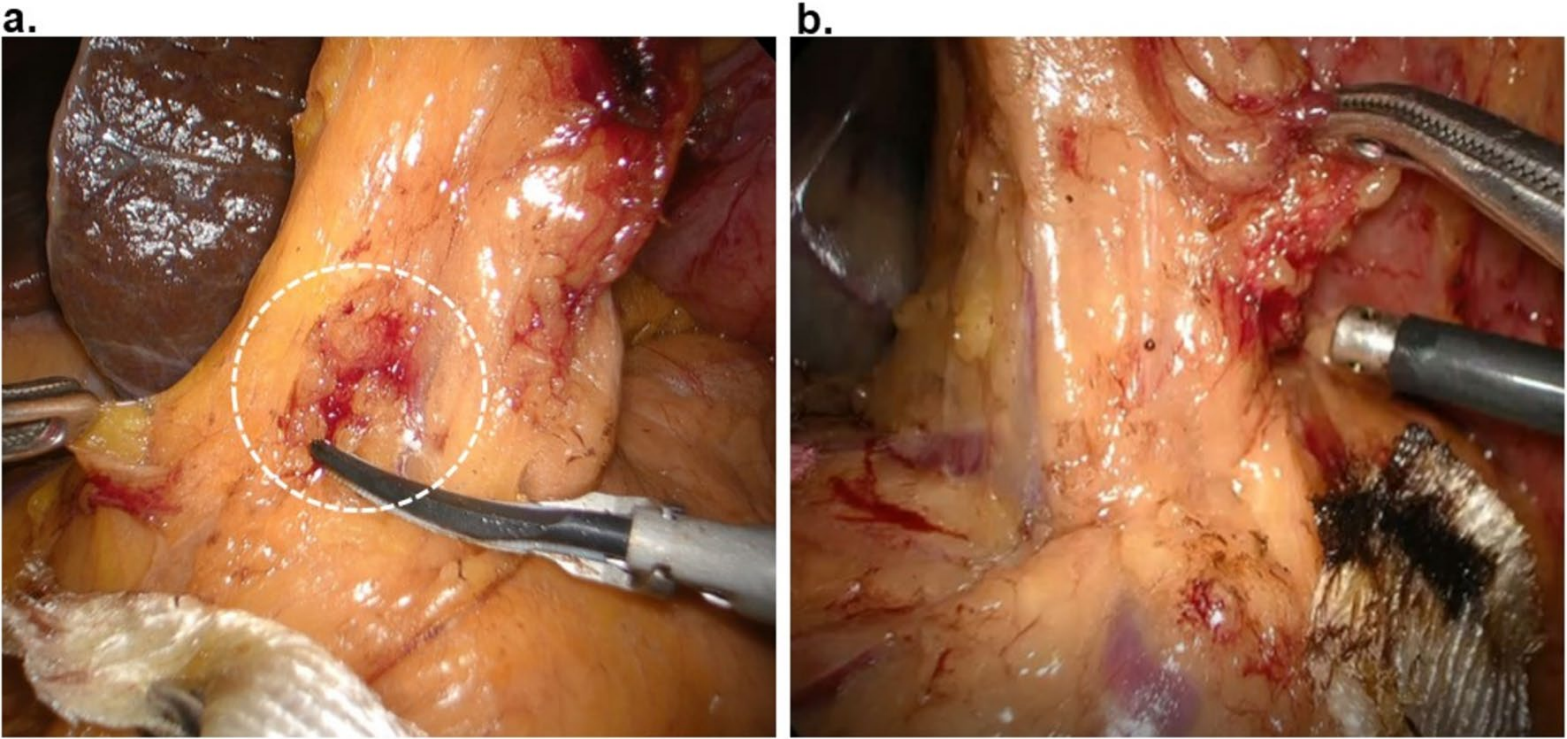
Representative intraoperative images of minor bleeding. (**a**) Hemostasis using an absorbable hemostatic agent. (**b**) Hemostasis using a coagulation system

Regarding the surgical outcomes, the investigated factors were operation time, operative blood loss, and incidence of postoperative complications. Regarding the intraoperative complications, we defined all intraoperative events that required hemostatic procedures using a coagulator or absorbable hemostatic gauze as minor bleeding (Fig. 3). Postoperative complications were classified in accordance with the Japan Clinical Oncology Group Postoperative Complications criteria with the general grading rules of the Clavien–Dindo classification system [6, 7]. POPF was defined as follows: grade 1: concentration of the drain amylase > three times the upper limit; grade 2: requiring pharmacological intervention; grade 3: requiring surgical intervention; and grade 4: requiring intensive care unit treatment. We defined the operative duration of No. 6 LND from the point when taking down the transverse mesocolon was finished to identify the RGEV to the point when the RGEV was divided by clipping.

This study involved two trainees who had over 10 years of experience as surgeons with board certification by the Japan Society of Surgery. This study also involved two specialists qualified by the Endoscopic Surgical Skill Qualification System of the Japan Society for Endoscopic Surgery; one or the other of these surgeons participated in the surgery for all cases.

### Statistical analysis

All statistical analyses were performed using JMP 8.0.1 (SAS, Cary, NC, USA). The chi-squared test and Mann–Whitney U test were used to compare the clinical values. *P* < 0.05 was defined as statistically significant.

## Results

### Prediction of difficulty in No. 6 LND using the CT images

Among the 15 anatomical parameters, the horizontal distance between the RGEV and the RGEA in the coronal plane (*p* = 0.04), the slope distance between the RGEV and the RGEA in the MIP image (*p* = 0.02), and the horizontal distance between the points of the upper edge of the pancreas and the root of the RGEV in the MIP image (*p* = 0.005) were significantly correlated with the intraoperative difficulty of No. 6 LND (Table 1).

Validation was subsequently performed for the top two parameters and the patients’ characteristics using the full patient dataset (Table 2). We also investigated the presence of a PPH as previously reported by Kobayashi et al. [4]. The presence of a PPH (*p* = 0.04), the slope distance between the RGEV and RGEA in the MIP image (*p* < 0.0001), and the horizontal distance between the points of the upper edge of the pancreas and the root of the RGEV in the MIP image (*p* < 0.001) were significantly correlated with the intraoperative difficulty of No. 6 LND. The most significant parameter, the slope distance between the RGEV and the RGEA in the MIP image (V–A length) was adopted as the prediction method of the difficulty of No. 6 LND. Because the average V–A length was 19.8 mm, the cutoff value was set at 20 mm. When the prediction of No. 6 LND difficulty was performed with a V–A length ≥ 20 mm, the sensitivity and specificity were 81% and 80%, respectively.

**Table 2:** Validation results for predicting the difficulty of No. 6 LND

Variables	Difficulty (-) (*n* = 36)	Difficulty (+) (*n* = 21)	p-value
Age (years)	23 / 13	10 / 11	0.23
Sex (men/women)	73.1 ± 10.2	72.2 ± 8.7	0.93
BMI	22.3 ± 4.7	23.6 ± 3.7	0.18
PPH (- / +)	34 / 2	15 / 5	**0.02**
**MIP**			
Root of RGEV – root of RGEA: SD	15.9 ± 4.1	26.9 ± 7.7	**< 0.0001**
Root of RGEV – Upper edge of pancreas: HD	15.5 ± 6.8	23.8 ± 9.3	**< 0.001**

*LND* lymph node dissection, *BMI* body mass index, *PPH* process of the pancreas head, *RGEV* right gastroepiploic vein, *RGEA* right gastroepiploic artery, *SD* slope distance, *HD* horizontal distance

The bold values means significant difference (p<0.05)

### Analysis of the risk factors for POPF

The risk factors for POPF were investigated in patients who had undergone LDG (Table 3). The incidence of POPF was significantly associated with longer operative time (248 ± 60 vs. 287 ± 39, *p* = 0.04). Additionally, the POPF rate was significantly higher in patients with difficult No. 6 LND than that in patients without difficulty (26.5% vs. 64.3%, respectively; *p* = 0.01). Sex, age, body mass index, blood loss, and minor bleeding during No. 6 LND were not associated with the incidence of POPF.

**Table 3:** Analysis of the risk factors for POPF in LDG patients

Variables	POPF (-) (*n* = 34)	POPF (+) (*n* = 14)	p-value
Sex (men / women)	19 / 15	9 / 5	0.59
Age (< 65 / ≧65)	3 / 31	4 / 10	0.09
BMI (< 25 / ≧25)	25 / 9	12 / 2	0.35
Operative time (min)	248 ± 60	287 ± 39	**0.04**
Blood loss (ml)	24.3 ± 43.8	9.8 ± 13.9	0.81
Minor bleeding during No.6 LND (- / +)	22 / 12	5 / 9	0.07
Difficulty (- / +)	25 / 9	5 / 9	**0.01**

*POPF* postoperative pancreatic fistula, *LDG* laparoscopic distal gastrectomy, *BMI* body mass index, *LND* lymph node dissection

The bold values means significant difference (p<0.05)

### Surgical outcomes of LDG performed by qualified surgeons, without preoperative No. 6 LND difficulty evaluation

Table 4 shows the surgical outcomes of LDG performed by qualified surgeons, without preoperative evaluation of the difficulty of No. 6 LND. The rate of intraoperative minor bleeding in the patients with difficult No. 6 LND was 75%, which was significantly higher than that in the patients without difficulty (18.4 vs. 75.0%, *p* = 0.01). The operative duration required for No. 6 LND in the patients with difficulty was longer than, but not significantly different from, that in the patients without difficulty (8.5 ± 4.4 vs. 13.7 ± 12.4, *p* = 0.06). The incidence of POPF tended to be higher in the patients with difficulty than that in the patients without difficulty (18.2 vs. 50.0%, *p* = 0.08), although the result was not significantly different. Moreover, the median drain amylase concentration on postoperative day 3 in the patients with difficulty of No. 6 LND also tended to be higher than that in the patients without difficulty (194 ± 62 vs. 613 ± 408, *p* = 0.11).

**Table 4:** Surgical outcomes of LDG performed by qualified surgeons, without preoperative evaluation of No. 6 LND difficulty

Variables	Difficulty (-) (*n* = 22)	Difficulty (+) (*n* = 8)	p-value
Operative duration of #6 LND (min.)	8.5 ± 4.4	13.7 ± 12.4	0.06
Minor bleeding (- / +)	16 / 6	2 / 6	**0.01**
Drain amylase concentration 1 POD (IU/L)	970 ± 213	880 ± 247	0.41
Drain amylase concentration 3 POD (IU/L)	194 ± 62	613 ± 408	0.11
**Postoperative pancreatic fistula**			
Total (- / +)	18 / 4	4 / 4	0.08
Grade (1 / 2, 3)	4 / 0	3 / 1	0.09

*LDG* laparoscopic distal gastrectomy, *LND* lymph node dissection, *POD* postoperative day

The bold values means significant difference (p<0.05)

### Surgical outcomes of LDG performed by trainees, with preoperative evaluation

Table 5 shows the surgical outcomes of LDG performed by trainees, with preoperative evaluation of the difficulty of No. 6 LND. The rate of intraoperative minor bleeding in the patients with difficulty of No. 6 LND was 80%, which was significantly higher than that in the patients without difficulty (25.0 vs. 80.0%, *p* = 0.01). The operative duration and drain amylase concentration (1 POD and 3 POD) were not significantly different between patients with vs. without difficulty of No. 6 LND (*p* = 0.93, *p* = 0.59 and *p* = 0.78, respectively). The incidence of POPF tended to be higher in patients with difficulty than that in patients without difficulty (12.5 vs. 50.0%, *p* = 0.09), although the difference was not significantly different. The incidence of POPF > grade 2 did not differ between the difficulty groups (12.5 vs. 20.0%, *p* = 0.67).

**Table 5:** Surgical outcomes of LDG performed by trainees, with preoperative evaluation of No. 6 LND difficulty

Variables	Difficulty (-) (*n* = 8)	Difficulty (+) (*n* = 10)	p-value
Operative duration of #6 LND (min.)	17.1 ± 13.1	16.7 ± 7.3	0.93
Minor bleeding (- / +)	6 / 2	2 / 8	**0.01**
Drain amylase concentration 1 POD (IU/L)	7690 ± 282	1053 ± 554	0.59
Drain amylase concentration 3 POD (IU/L)	116 ± 55.5	1616 ± 1490	0.78
**Postoperative pancreatic fistula**			
Total (- / +)	7 / 1	5 / 5	0.09
Grade (1 / 2, 3)	0 / 1	3 / 2	0.67

*LDG* laparoscopic distal gastrectomy, *LND* lymph node dissection, *POD* postoperative day

The bold values means significant difference (p<0.05)

### Comparison of surgical outcomes in LDG between qualified surgeons and trainees

Table 6 shows the comparison of surgical outcomes in LDG between qualified surgeons and trainees. The operative duration required for No. 6 LND performed by trainees tended to be longer than that required by the qualified surgeons (15.4 ± 9.7 vs. 10.4 ± 8.5, *p* = 0.07); however, the difference was not significant. The rate of intraoperative minor bleeding and the incidence of POPF did not differ between the two groups of surgeons (55.6 vs. 40.0%, *p* = 0.29 and 33.3 vs. 26.7%, *p* = 0.62, respectively). Moreover, the median drain amylase concentration on postoperative days 1 and 3 was not different between the groups of surgeons (991 ± 309 vs. 933 ± 157, *p* = 0.80 and 1229 ± 1118 vs. 291 ± 110, *p* = 0.41, respectively).

Table 7 shows the comparison of surgical outcomes in LDG with difficulty between qualified surgeons and trainees. Even in the cases with difficulty, the surgical outcomes including the operative duration required for No. 6 LND (16.7 ± 7.3 vs. 13.7 ± 12.4, *p* = 0.35), the rate of intraoperative minor bleeding (80.0 vs. 75.0%, *p* = 0.41) and the incidence of POPF (50.0 vs. 50.0%) did not differ between the two groups of surgeons. Preoperative evaluation of the difficulty of No. 6 LND enables trainee to perform LDG safely even in the case with difficulty.

**Table 6:** Comparison of surgical outcomes in LDG between qualified surgeons and trainees

Variables	Trainee (*n* = 18)	Qualified surgeon (*n* = 30)	p-value
Operative duration of #6 LND (min.)	15.4 ± 9.7	10.4 ± 8.5	0.07
Minor bleeding (- / +)	8 / 10	18 / 12	0.29
Drain amylase concentration 1 POD (IU/L)	991 ± 309	933 ± 157	0.80
Drain amylase concentration 3 POD (IU/L)	1229 ± 1118	291 ± 110	0.41
**Postoperative pancreatic fistula**			
Total (- / +)	12 / 6	22 / 8	0.62
Grade (1 / 2, 3)	3 / 3	7 / 1	0.26

*LDG* laparoscopic distal gastrectomy, *LND* lymph node dissection, *POD* postoperative day

**Table 7:** Comparison of surgical outcomes in LDG with difficulty between qualified surgeons and trainees

Variables	Trainee (*n* = 10)	Qualified surgeon (*n* = 8)	p-value
Operative duration of #6 LND (min.)	16.7 ± 7.3	13.7 ± 12.4	0.35
Minor bleeding (- / +)	2 / 8	2 / 6	0.41
Drain amylase concentration 1 POD (IU/L)	1053 ± 554	880 ± 247	0.62
Drain amylase concentration 3 POD (IU/L)	1616 ± 1490	613 ± 408	0.87
**Postoperative pancreatic fistula**			
Total (- / +)	5 / 5	4 / 4	N.D
Grade (1 / 2, 3)	3 / 2	3 / 1	0.67

*LDG* laparoscopic distal gastrectomy, *LND* lymph node dissection, *POD* postoperative day, *N.D* not detected

## Discussion

In this study, we revealed the usefulness of preoperative evaluation of the difficulty of No. 6 LND for trainees performing LG. LG requires a long learning curve of 40–100 cases because the procedures are relatively complicated for trainees, such as multiple stages in the LND and resection of several major blood vessels [8]. Various training systems for young surgeons have been reported as well as standardization of the procedure in laparoscopic surgery [9, 10]. We previously reported the usefulness of preoperative simulation for gastrectomy [11]. We routinely perform detailed preoperative simulations that include three-dimensional simulation for all surgical cases undergoing hepatectomy, pancreatectomy, colorectal surgery, and gastrectomy. Regarding preoperative three-dimensional simulation for gastrectomy, this approach enables trainees to perform LG safer than without the simulation [11]. However, we have often encountered difficulty in No. 6 LND during the operation. Because the No. 6 lymph node directly faces the pancreas, the difficulty of No. 6 LND correlates with the risk of pancreatic injury resulting in POPF. To overcome this difficulty, preoperative evaluation methods should be established.

Previous reports identified the risk factors for POPF related to LG as sex (male), age, obesity, and operative time [12, 13]. POPF is mainly caused by intraoperative procedures, such as thermal injury caused by energized devices [14, 15], blunt injury from compression and retraction, and bleeding from the pancreas [16]. Additionally, the technical difficulties of the operative procedures differ for each case depending on an individual’s anatomical characteristics. There were a few reports to predict the incidence of POPF using a preoperative CT image. One focused on the anatomical feature calling “process of the pancreas head (PPH) using preoperative CT images [4]. To identify the PPH was useful in preventing POPF. The others focused on the position of the pancreas. The characteristic of anatomical pancreas position varies widely and some features related with POPF. Migita et al. reported that the distance between the pancreatic body surface and the root of the common hepatic artery was identified as an independent predictor of POPF [17]. Kinoshita et al. also reported that the vertical length between the upper border of pancreas and the root of left gastric artery in the sagittal direction could predict the risk of POPF in LG but not in open gastrectomy [18]. This suggests that the risk factors for POPF in LG may differ from those after OG. Kumagai et al. reported that the angle between the upper border of the pancreas to the root of the celiac artery related to drain amylase concentration on postoperative days 1 [19]. To the best of our knowledge, this study is the first report to clearly show the benefit to predict the difficulty in No. 6 LND for preventing POPF. In the present study, we found that POPF was significantly associated with operative time and the difficulty of No. 6 LND. The rate of intraoperative minor bleeding was significantly higher in difficult No. 6 LND cases that that in non-difficult cases, even when LG was performed by certified surgeons. Furthermore, this difficulty can be easily predicted by V–A length using preoperative CT images, with high sensitivity and specificity (81% and 80%, respectively). Additionally, even in difficult No. 6 LND cases, trainees could perform LDG safely compared with certified surgeons. The preoperative recognition of No. 6 LND difficulty using CT images can reduce the risk of POPF by raising awareness to potential difficulties and enabling surgeons to take appropriate preventive measures. Since preoperative evaluation of the difficulty of No. 6 LND enables trainee to perform LDG safely even in the case with difficulty, our prediction may contribute to improve surgical outcomes including long-term outcomes. It is necessary to increase the number of patients and long-term follow-up in the future.

This study has some limitations. First, this was a retrospective study resulted in the risk of selection bias. Second, since this study was performed in a single institution, the results may not be generalizable. Further studies enrolling a larger number of patients in multi-institution are needed. Finally, the results were based on the standardization of the surgical procedure in our institution which may therefore only be valid for patients in other hospitals using the same procedure. Nevertheless, our results showed that right gastroepiploic V–A length was easily measured using preoperative CT images, and that this length was a reliable predictive marker of No. 6 LND difficulty in LDG. Moreover, it is important for trainees to perform detailed preoperative simulations to understand and visualize the intraoperative surgical view.

## Conclusions

Our study newly defines the characteristic of the dissection along the RGEV requiring > 2 cm from the root of the RGEV as difficulty of No. 6 LND. The No. 6 LND difficulty is related to minor bleeding during LND and which is considered to cause POPF. No. 6 LND difficulty was easily predicted using CT images. The recognition of difficulty can reduce the risk of POPF by raising awareness to potential difficulties and enabling a safe surgical procedure for trainees.

## Data Availability

The datasets used and/or analyzed during the current study are available from the corresponding author on reasonable request.

## Abbreviations

LG: Laparoscopic gastrectomy
LND: Lymph node dissection
POPF: Postoperative pancreatic fistula
CT: Computed tomography
RGEV: Right gastroepiploic vein
LDG: Laparoscopic distal gastrectomy
RGEA: Right gastroepiploic artery
